# Inferior exten filtering bleb formation after laser goniopuncture in a patient with conjunctivochalasis

**DOI:** 10.1016/j.ajoc.2026.102526

**Published:** 2026-01-27

**Authors:** Abdulrahman Alhazmi, Fahad Alharthi, Jumanah Qedair

**Affiliations:** a Glaucoma Division, King Khaled Eye Specialist Hospital and Research Center, Riyadh, Saudi Arabia; b College of Medicine, King Saud bin Abdulaziz University for Health Sciences, Jeddah, Saudi Arabia

**Keywords:** Conjunctivochalasis, Deep sclerectomy, Filtering bleb, Glaucoma surgery, Laser goniopuncture

## Abstract

**Purpose:**

Inferior extension of filtering blebs after glaucoma surgery is rare. Conjunctivochalasis, an age-related redundancy of the conjunctiva, may influence bleb morphology but is often overlooked. This report describes an inferiorly extended filtering bleb following deep sclerectomy and subsequent Nd:YAG laser goniopuncture in a patient with pre-existing conjunctivochalasis.

**Methods:**

A 66-year-old man with advanced primary open-angle glaucoma underwent combined phacoemulsification, deep sclerectomy, and intraoperative mitomycin C in the right eye. Initial recovery was uneventful, with IOP at 15 mmHg and a mildly elevated bleb.

**Results:**

At six weeks, bleb flattening and an IOP rise to 19 mmHg prompted Nd:YAG laser goniopuncture. Two weeks later, a diffuse, hypovascular bleb extended inferiorly into the fornix, corresponding to an area of conjunctivochalasis. IOP was 9 mmHg, with no hypotony, leak, or symptoms. The bleb regressed spontaneously over one month, stabilizing superiorly with IOP at 13 mmHg without medications.

**Conclusions:**

This case suggests that conjunctivochalasis may act as a low-resistance pathway for aqueous diffusion after increased outflow from goniopuncture, leading to atypical bleb spread. While self-limiting here, such morphology could have implications for comfort, contact lens use, or hypotony risk. Pre-existing conjunctival redundancy may influence postoperative bleb morphology and should be considered in surgical planning and follow-up after bleb-forming glaucoma surgeries.

## 1 Introduction

Bleb-forming glaucoma surgeries, such as deep sclerectomy create a controlled pathway for aqueous humor to drain into the subconjunctival space, forming a filtering bleb that lowers intraocular pressure (IOP).^1^^,^^2^ While a degree of variability in bleb morphology is expected, unusual presentations such as extensive inferior extension are rare and warrant closer evaluation.^3^

The final morphology and extent of a filtering bleb are influenced by several factors, including surgical technique, the use of antimetabolites, and the individual's healing response.^3^, ^4^, ^5^ Conjunctivochalasis is one of the anatomical factors determining the morphology, which is an age-related condition characterized by redundant, loose conjunctival tissue, most commonly found in the inferior fornix.^6^^,^^7^ Although frequently asymptomatic, this conjunctival laxity may alter postoperative fluid dynamics by providing a potential low-resistance pathway for aqueous diffusion.^8^^,^^9^

This case report describes an unusual inferior extension of a filtering bleb following deep sclerectomy and subsequent Nd:YAG laser goniopuncture in a patient with pre-existing conjunctivochalasis. We suggest that the conjunctivochalasis acted as a conduit for the aqueous humor after outflow was enhanced by goniopuncture, leading to a transient, self-limiting bleb in an atypical location. This case highlights the potential for pre-existing conjunctivochalasis to influence postoperative bleb morphology, a consideration that may be relevant in surgical planning and follow-up for bleb-forming glaucoma surgeries.

## 2 Case description

A 66-year-old male with bilateral primary open-angle glaucoma (POAG) presented for surgical intervention in the right eye due to progressive visual field loss despite maximal medical therapy (timolol–dorzolamide twice daily, travoprost at bedtime, and brimonidine twice daily). His IOP in the right eye was 15 mmHg, with advanced glaucomatous optic nerve cupping (cup-to-disc ratio 0.9 with inferior and superior notching) and severe visual field constriction. Preoperatively, slit-lamp examination revealed temporal and inferior conjunctival redundancy suggestive of conjunctivochalasis in both eyes.

The patient underwent uncomplicated phacoemulsification with posterior chamber intraocular lens implantation combined with deep sclerectomy and intraoperative mitomycin C application (0.2 mg/mL for 2 minutes) in the right eye. On the first postoperative day, the IOP was low (not measurable by Goldmann applanation), and the eye was digitally soft, without clinical signs of hypotony maculopathy or choroidal detachment. Early recovery was otherwise uneventful, with a mildly elevated bleb and an IOP of 15 mmHg at the subsequent follow-up.

**Fig. 1:**
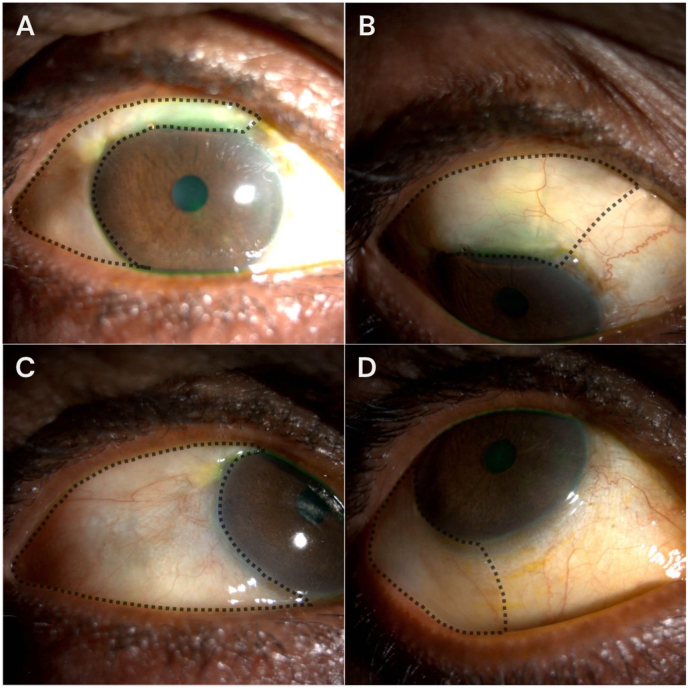
Slit-lamp photographs of the right eye two weeks after Nd:YAG laser goniopuncture **(A**–**D)**. **(A)** Primary position showing a diffuse, low-lying superior filtering bleb with inferior extension. **(B)** Downward gaze exposing the superior bulbar conjunctiva and superior filtering bleb. **(C)** Upward gaze demonstrating inferior extension of the filtering bleb over the inferior bulbar conjunctiva, associated with conjunctivochalasis. **(D)** Inferior oblique view further illustrating the inferior extent of the filtering bleb. Dotted lines delineate the margins of the filtering bleb.

At six weeks postoperatively, due to bleb flattening and an increase in IOP to 19 mmHg, Nd:YAG laser goniopuncture was performed using a Q-switched laser (spot size 5 μm, energy 8 mJ per pulse), with approximately six laser applications delivered to the trabeculo-Descemet's window to enhance aqueous outflow. Two weeks later, examination revealed an extensive, non-cystic, diffuse filtering bleb that extended inferiorly beyond the typical limbal area, reaching the inferior fornix (Fig. 1). The bleb was hypovascular, low-lying, and caused no discomfort or diplopia. IOP was 9 mmHg, and there was no evidence of bleb leak, hypotony maculopathy, or choroidal detachment. The location of bleb expansion corresponded to an area previously noted to have conjunctivochalasis.

**Fig. 2:**
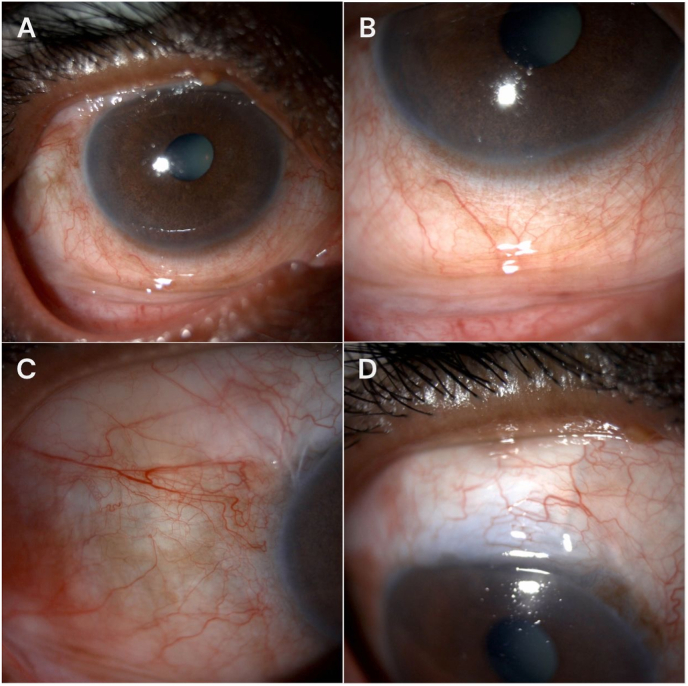
Slit-lamp photographs of the right eye one month after Nd:YAG laser goniopuncture **(A**–**D)**. **(A)** Primary position showing a low-lying superior filtering bleb without inferior extension. **(B)** Upward gaze highlighting the inferior bulbar conjunctiva, demonstrating regression of the previously extended inferior filtering bleb. **(C)** Higher-magnification view of the inferior bulbar conjunctiva confirming absence of inferior bleb extension. **(D)** Downward gaze showing stabilization of the filtering bleb within the superior quadrant.

The patient was observed without intervention, and over the following month, the bleb gradually decreased in size and stabilized superiorly (Fig. 2). Final IOP remained controlled at 13 mmHg with no glaucoma medications.

## 3 Discussion

This case illustrates a direct temporal relationship between Nd:YAG laser goniopuncture and the transient inferior extension of a filtering bleb in an eye with pre-existing conjunctivochalasis. We propose that the sudden augmentation of aqueous outflow following goniopuncture exploited the low-resistance pathway created by the redundant conjunctiva, leading to the atypical bleb morphology.

While conjunctivochalasis is a common, often incidental finding, its potential to influence postoperative fluid dynamics after glaucoma surgery is rarely considered.^6^ Here, we document it as a primary factor in directing bleb extension. Although the presentation was self-limiting in our patient, it may highlight that conjunctivochalasis is not merely a benign finding in the surgical context. It may predispose to dependent, mobile blebs with potential implications for hypotony risk, patient comfort, and contact lens tolerance.^7^^,^^10^

This report highlights conjunctivochalasis as a relevant anatomical variable in surgical planning and postoperative management. Recognizing its presence may allow surgeons to anticipate unusual bleb spread and counsel patients accordingly, especially when interventions like goniopuncture are planned.

## CRediT authorship contribution statement

**Abdulrahman Alhazmi:** Writing – review & editing, Writing – original draft, Validation, Resources, Project administration, Methodology, Investigation, Formal analysis, Data curation, Conceptualization. **Fahad Alharthi:** Writing – review & editing, Supervision, Resources, Conceptualization. **Jumanah Qedair:** Writing – review & editing, Writing – original draft, Validation, Software, Resources, Methodology, Data curation.

## Patient consent

Consent to publish this case report was obtained from the patient. This report does not contain any personal information that could lead to the identification of the patient.

## Claims of priority statement

After conducting a literature review in September 2025 through PubMed, Google Scholar, Cochrane, and Web of Science databases using the key words (Conjunctivochalasis; Deep sclerectomy; Filtering bleb; Glaucoma surgery; Laser goniopuncture), we did not find any prior reports of such an incidence in patients with conjunctivochalasis.

## Authorship

All authors attest that they meet the current ICMJE criteria for authorship.

## Funding

This research did not receive any specific grant from funding agencies in the public, commercial, or not-for-profit sectors.

## Declaration of competing interest

The authors declare that they have no known competing financial interests or personal relationships that could have appeared to influence the work reported in this paper.
