# Corrigendum to “Inferior extension of a filtering bleb following laser goniopuncture in a patient with conjunctivochalasis” [Am J Ophthalmol Case Rep 41 (2026) 102526]

**DOI:** 10.1016/j.ajoc.2026.102538

**Published:** 2026-02-09

**Authors:** Abdulrahman Alhazmi, Fahad Alharthi, Jumanah Qedair

**Affiliations:** a Glaucoma Division, King Khaled Eye Specialist Hospital and Research Center, Riyadh, Saudi Arabia; b College of Medicine, King Saud Bin Abdulaziz University for Health Sciences, Jeddah, Saudi Arabia

• The authors regret that the following text was included in error in the Abstract (Methods) section: "combined phacoemulsification…"
• The authors regret that the following text was included in error in the Case Description section: "phacoemulsification with posterior chamber intraocular lens implantation combined with…"

The authors would like to apologise for any inconvenience caused.

